# A descriptive study of healthcare-providers’ experiences with the use and quality of oxytocin for the prevention of post-partum hemorrhage in Nigeria: A nation-wide survey

**DOI:** 10.1371/journal.pone.0258096

**Published:** 2021-10-06

**Authors:** Chioma S. Ejekam, Florence M. Nyangara, Chimezie Anyakora, Jude Nwokike

**Affiliations:** 1 Department of Community Health, Lagos University Teaching Hospital, Lagos, Nigeria; 2 United States Pharmacopeial Convention (USP), Rockville, Maryland, United States of America; 3 Bloom Public Health, Utako, Abuja, Nigeria; Eberhard Karls University Tübingen: Eberhard Karls Universitat Tubingen, GERMANY

## Abstract

**Background:**

Oxytocin is recommended as an affordable and effective drug in the prevention of postpartum hemorrhage—one of the leading causes of maternal morbidity and mortality in low- and middle-income countries, however, there are concerns about its proper use and quality. This study builds on earlier work conducted in a South-Western state in Nigeria.

**Objective:**

The study assessed the knowledge around oxytocin, usage, storage practices and perceived quality of oxytocin used by healthcare providers that directly administer oxytocin for the prevention of postpartum hemorrhage across Nigeria.

**Methods:**

This was a descriptive cross-sectional study that surveyed a representative sample of 6,299 healthcare providers who offer obstetrics and gynecological services and recruited from 1,894 healthcare facilities in Public and Private sectors in 12 states across Nigeria. Data were collected using an electronic questionnaire, analyzed using SPSS, and presented in frequencies and percentages.

**Results:**

Only forty-six percent of respondents (52.8% in private; 40.0% in public sector) had proper knowledge that oxytocin storage is in the refrigerator. Proper knowledge also varied by professional cadre, doctors (71.2%); nurses (46.6%); Community Health Workers (28.4%) and by years of experience, less than 10 years (51.4%); more than 10 years (40.8%). Only 34% of the respondents (41% in private and 27.5% in public sector) reported good practices that oxytocin is stored in the refrigerator in their facilities. Most healthcare providers used oxytocin for prevention of PPH (77.9%). Oxytocin was also used for augmentation (66.7%) and induction of labor (52.6%). Half of respondents used above the WHO-recommended oxytocin dose of 10IU for prevention of PPH. Twenty-three percent of respondents reported experiencing oxytocin failure in PPH prevention of whom, 54.3% changed to another uterotonic and 37.1% doubled the dose of oxytocin for their patients.

**Conclusion:**

Our study findings should be used to establish clinical guidelines and trainings for healthcare providers to improve their knowledge and storage practices and use to safeguard the quality of these lifesaving medicines.

## Introduction

Nigeria has one of the highest maternal mortality ratio (914/100,000 live births) that is estimated to account for 19 percent of the global maternal deaths [1, 2]. Every day in Nigeria, about 145 women between the ages of 15 and 45 years die from preventable causes linked to pregnancy and childbirth [3, 4]. It is widely acknowledged that obstetric hemorrhage, especially postpartum hemorrhage (PPH), is one of the leading causes of maternal morbidity and mortality in low- and middle-income countries (LMICs) including Nigeria [5–7]. Nigeria’s maternal morbidity and mortality also varies by geopolitical zones, where the North East and North West zones have almost 10 and 6 times higher rates, respectively, than the South West zone; by state; and by rural/urban residence, where women in rural areas are at a higher risk of deaths than those in urban areas [8]. In 2019, the National Primary Healthcare Development Agency (NPHCDA) declared a state of emergency on both maternal and child health in Nigeria following the alarming statistics on the frequency of maternal and child deaths, especially at the primary healthcare level. NPHCDA committed to rapidly reduce these deaths by half by 2021 through interventions that target the primary healthcare and community levels [3]. The interventions include promoting access to quality care before, during, and after childbirth and focusing on preventable causes of maternal deaths during this period including prevention and treatment of PPH [4]. PPH is commonly defined as blood loss of 500 mL or more within 24 hours after birth. Indeed, PPH is associated with nearly one-quarter of the global maternal deaths and a contributor to long-term maternal morbidity and disability including shock and organ dysfunction [9, 10]. Fortunately, maternal mortality, morbidity and disability due to PPH are largely preventable.

According to the UN Commission Report in 2012, oxytocin is among the listed lifesaving commodities for women during pregnancy and childbirth within the continuum of care [11]. The World Health Organization (WHO) recommends the use of prophylactic uterotonics during the third stage of labor to reduce the incidence of PPH [10] and oxytocin for the prevention and treatment of PPH [12]. Oxytocin is also included both in the “WHO Model List of Essential medicines” and the Essential medicines’ list for Nigeria [11, 13]. According to the latest WHO recommendations [14], oxytocin to prevent PPH in women having a vaginal birth should be administered immediately after delivery by intramuscular or slow intravenous bolus injection Oxytocin should be administered by intramuscular injection, intravenous infusion, or intravenous bolus injection and it should be administered by a skilled and trained healthcare provider [15, 16]. In addition, oxytocin is known to be sensitive to heat on exposure. Therefore, to ensure the longest possible life, oxytocin should be maintained throughout transport and storage at 2–8°C [17]. Previously, some manufacturers have had different storage recommendations for oxytocin and these have resulted to conflicting storage practices and consequent quality assessment of this product [18]. For consistency of information and practice, WHO/UNICEF/UNFPA issued a joint statement after exhaustive review of current evidence around oxytocin and urged partners to strongly ensure that oxytocin is labeled to clearly indicate storage and transport requirements at 2–8°C [17].

Although WHO recommends oxytocin as an effective and affordable drug in the prevention and treatment of PPH, there have been concerns about its quality in LMICs. Previous studies have documented that a significant proportion of oxytocin injections available in LMICs, including in Nigeria, fail to meet acceptable international quality standards, which compromises their effectiveness [19–22]. These studies have suggested that poor-quality oxytocin could be a result of either substandard manufacturing, improper transportation and storage conditions along the supply chain, or a combination of both [20].

### Research context

Oxytocin requires cold chain supply for it to maintain its effectiveness, however, most facilities in LMICs especially those at the primary healthcare level have no refrigerators or reliable electricity supply [23]. There is growing evidence showing that when poor-quality oxytocin is used and it fails to prevent and manage PPH adequately, healthcare providers result into increasing doses of oxytocin or changing to other uterotonic medicines in efforts to stop severe blood loss [23]. Poor-quality medicines, including oxytocin can result in untimely maternal death, negatively influence public confidence in the healthcare system, lead to financial, psychological, and social impact, and economic losses to families [24, 25].

Further evidence indicates that healthcare providers’ knowledge on the proper storage conditions and use of oxytocin is critical to ensuring the quality of medicines [26–29]. In 2017, a pilot study on clinical experiences of healthcare providers with the use of oxytocin in Lagos State Nigeria found that only 52 percent of respondents knew the proper storage conditions for oxytocin, and more than 41 percent administered more than the WHO-recommended dose of oxytocin in the prevention of PPH [27]. Previous studies conducted in LMICs have also reported inappropriate clinical use of oxytocin, including administration of varying dosages from the recommended doses [24, 26, 28, 30]. Findings from these studies have also generated questions on whether the use of oxytocin above the WHO recommended dose is a reflection of limited knowledge among healthcare providers and their suspicion that the oxytocin medicine they are using are not effective, an indication of poor quality. The pilot study conducted in Lagos—which is the richest state in Nigeria revealed poor knowledge of oxytocin storage and inappropriate use both intra-partum and post-partum. There was urgent need for additional research involving a large and representative sample of healthcare providers that directly administer oxytocin to clients to assess and understand their knowledge, usage, storage practices, and perceived quality and effectiveness of oxytocin in the prevention of PPH across the six geopolitical zones of Nigeria. In addition, the current study focused on the use of oxytocin for the prevention of PPH, while the Lagos pilot study included both the intra-partum and post-partum (prevention of post-partum hemorrhage) use of oxytocin. The current study assessed the knowledge around oxytocin, usage, storage practices and perceived quality of oxytocin used by healthcare providers that directly administer oxytocin for the prevention of postpartum hemorrhage across Nigeria.

## Materials and methods

### Study design

This was a descriptive cross-sectional study design conducted in early 2019.

### Study population

We surveyed a sample of 6,299 healthcare providers who deliver babies [doctors, nurses, community health workers (CHWs) who are either Community Health Officers (CHO) or Community Health Extension Workers (CHEW)] from 1,894 healthcare facilities that offer obstetrics and gynecological services in 12 States across the six geo-political zones of Nigeria. Nigeria has 36 states and 774 local government areas (LGAs) unevenly spread across its six geopolitical zones. The sampling frame used for the current study to select states included a list of 35 out of 36 states of Nigeria. Lagos state was not included in the study.

### Sample size determination

The sample size for the study population was determined using Cochran’s formula [31]. Making provisions for a 30-percent nonresponse rate to a self-administered questionnaire, the minimum estimated sample size for a state in one geopolitical zone was 488. The estimated total sample size was 5,856 in 12 states. In states where the allotted sample size could not be met, more respondents were selected from the other states within the same zone. The final total sample of 6,299 healthcare providers, consisting of doctors, nurses/midwives and CHOs were successfully interviewed across Nigeria.

### Selection of respondents

The sample of respondents consisted of practicing doctors and nurses/midwives, Community Health Workers (CHWs) including CHOs and CHEWs registered in public or private facilities that offer obstetrics and gynecological services and use oxytocin in their practices. The CHWs were selected to participate in facilities without skilled staff (doctors or nurses/mid-wives) for the current study, unlike the Lagos pilot study sample that only included doctors and nurses/mid-wives. Traditional birth attendants were excluded, as they are not registered to use oxytocin in their practice in Nigeria.

To ensure representation, the selection of study respondents was based on the relative number of healthcare providers who met the inclusion criteria per facility and proportion of doctor to nurse/mid-wife ratio. The doctor to nurse/mid-wife ratio of 1:2 was used to select respondents per facility. A maximum of six respondents were selected per facility even in large public healthcare facilities.

A multistage sampling design was used to select states from each geopolitical zone, LGAs, healthcare facilities, and then respondents. Stage 1, two states were selected from each geopolitical zone by simple random sampling for a total of 12 states. The states selected were Ebonyi, Imo, Delta, Akwa-Ibom, Oyo, Ekiti, Nasarawa, Niger, Bauchi, Gombe, Jigawa. Stage 2, four LGAs were randomly selected from each selected state, except, LGAs that host the tertiary hospitals which were purposively selected. Stage 3, facilities from each selected LGAs that were registered as public and private/non-profit healthcare facilities that offer obstetrics and gynecological services. Public sector lists of facilities were organized by tertiary, secondary, and primary levels per LGAs and a separate list of registered private hospitals (including nonprofit) per LGA where available were obtained from the State Ministries of Health. Facilities were selected using systematic random sampling per LGA. If a selected facility no longer existed or did not offer gynecological services, the next one on the list was selected. A total of 1,894 health facilities were selected: Public sector (52%), Private sector (47%), and Nonprofit organizations (<1%). Stage 4, a sample of healthcare providers who deliver babies in the selected public or private facilities at the primary, secondary, or tertiary level were included in this study.

### Recruitment and training of data collectors and field supervisors

Thirty-six research assistants with at least a Bachelor of Science in a health or medical science-related field were recruited and trained as data collectors for this study. Their selection was based on whether they were residents in their respective state; had good communication skills; and were fluent in English, Pidgin, and the local language specific to that state. Four supervisors with experience in public health were recruited, trained, and assigned to supervise three states each during the period of data collection. The research team was trained to understand the study protocol, the questionnaires and how to upload the data to the electronic version of the questionnaire (kobotoolbox). Every member of the research team had a smartphone to enable access to an electronic copy of the questionnaire. The supervisors were in the field with their assigned research assistants in their respective states throughout the period of data collection to ensure the validity and quality of data collected.

### Questionnaire development

The questionnaire was adapted from the pilot Lagos study version, which was developed following a literature review, expert reviews, and opinions [27]. The questionnaire included multiple response questions aimed to capture respondents’ perspectives and different ways oxytocin is used and stored in practice. The questionnaire was pretested among 120 clinicians (doctors and nurses/mid-wives) who met the inclusion criteria, showed willingness to participate, and were from facilities in the states not included in the study. Data were collected using the final pretested self-administered questionnaire. The questionnaire had three sections. Section A collected information on respondents’ sociodemographic characteristics, section B assessed occupational history, and section C assessed clinical experience with oxytocin use.

### Outcomes

The primary outcomes of the study were proper knowledge and practice of oxytocin storage, proper use of oxytocin dosage for PPH prevention in the past one year and perceived effectiveness of oxytocin injections used in clinical practice by healthcare providers. The Covariates were age, sex, professional cadre of respondents, their years of work experience in current role of delivering babies, type of facility, and States.

### Data quality assurance

Data quality were assured by providing thorough and proper training of the research team members, pretesting of the data collection tools, both the paper questionnaire and electronic version and also ensuring that field supervisors conducted field check-ins with their research assistants throughout the data collection period. Data were uploaded daily from the paper questionnaire into an electronic version. Each completed questionnaire was crosschecked for quality by the supervisor. The principal investigator monitored daily data collection uploaded to the server (the kobotoolbox) and provided feedback to supervisors where necessary. This two-step data validation process continued throughout the data collection period. Spot checks were conducted to randomly selected states by the principal investigator to validate the data during fieldwork.

### Data analysis

Data analysis were done using the Statistical Package for Social Sciences (SPSS) software, version 21 and presented in frequency tables. Categorical variables including multiple response variables were summarized using simple proportions. Mean and standard deviation were used to summarize quantitative variables that were normally distributed, while median and interquartile ranges were used for those that were found not to be normally distributed.

### Ethical considerations

Ethical approval was obtained from the National Health Research Ethics Committee of Nigeria (NHREC) with assigned no. NHREC/01/01/2007. A signed informed consent was obtained from each respondent before interviewing them.

## Results

A total of 6,299 healthcare providers were surveyed between January and March 2019. Table 1 shows the background characteristics of the respondents,18.7 percent were doctors, 53.4 percent were nurses/midwives, and 27.9 percent were CHWs. Slightly more than one-third (35.7%) of respondents were between 31–40 years old. On average, doctors were older with a mean age of 39.9 ±10.3 years, 36.7 ±10.3 years for nurses/midwives; and 36.9 ±8.9 years for CHWs. Most of the healthcare providers were female (77.9%). Years of experience ranged from 8 to 10 years (9 years for doctors, 8 years for nurses/‌midwives, and 10 years for CHWs).

**Table 1:** Background characteristics of respondents.

Background Characteristics		Profession cadre							
		Doctor		Nurse/Midwife		CHW		Overall	
		N	%	N	%	N	%	N	%
	Overall	***1179***	***18*.*7***	***3362***	***53*.*4***	***1758***	***27*.*9***	***6299***	***100*.*0***
**Sex**	Male	984	*83*.*5*	194	*5*.*8*	216	*12*.*3*	1394	***22*.*1***
	Female	195	*16*.*5*	3168	*94*.*2*	1542	*87*.*7*	4905	***77*.*9***
**Age group**	< = 34yrs	437	*37*.*1*	1608	*47*.*8*	714	*40*.*6*	2759	***43*.*8***
	35-54yrs	623	*52*.*8*	1558	*46*.*3*	1000	*56*.*9*	3181	***50*.*5***
	>55yrs	119	*10*.*1*	196	*5*.*8*	44	*2*.*5*	359	***5*.*7***
**Years of experience**	<10yrs	625	*53*.*0*	1789	*53*.*2*	758	*43*.*1*	3172	***50*.*4***
	= >10yrs	554	*47*.*0*	1573	*46*.*8*	1000	*56*.*9*	3127	***49*.*6***
**Health facility type**	Public	398	*33*.*8*	1581	*47*.*0*	1295	*73*.*7*	3274	***52*.*0***
	Private	781	*66*.*2*	1781	*53*.*0*	463	*26*.*3*	3025	***48*.*0***

Table 2 shows that majority of respondents (74.0%) reported that they have had training on the use or storage of oxytocin. Eighty-eight percent of doctors, 76.1 percent of nurses/midwives and 60.6 percent of CHWs have had training on the use or storage of oxytocin. A marginally higher proportion of healthcare providers in private healthcare facilities (78.3%) reported having received training on the use or storage of oxytocin compared to those in public healthcare facilities (70.1%).

**Table 2:** Respondents’ assessment of training about oxytocin.

Background Characteristics		Ever had training about oxytocin?						
		Yes		No		Not sure		Total
		N	%	N	%	N	%	N
**Sex**	Male	1150	82.5	200	*14*.*3*	44	*3*.*2*	1394
	Female	3513	71.6	1224	*25*.*0*	168	*3*.*4*	4905
**Profession**	Doctor	1037	88.0	106	*9*.*0*	36	*3*.*1*	1179
	Nurse/midwife	2560	76.1	709	*21*.*1*	93	*2*.*8*	3362
	CHW	1066	60.6	609	*34*.*6*	83	*4*.*7*	1758
**Age group**	< = 34yrs	2075	75.2	593	*21*.*5*	91	*3*.*3*	2759
	35-54yrs	2297	72.2	771	*24*.*2*	113	*3*.*6*	3181
	>55yrs	291	81.1	60	*16*.*7*	8	*2*.*2*	359
**Years of experience**	<10yrs	2400	75.7	670	*21*.*1*	102	*3*.*2*	3172
	= >10yrs	2263	72.4	754	*24*.*1*	110	*3*.*5*	3127
**Health facility type**	Public	2294	70.1	856	*26*.*1*	124	*3*.*8*	3274
	Private	2369	78.3	568	*18*.*8*	88	*2*.*9*	3025
	**Total**	***4663***	***74*.*0***	***1424***	***22*.*6***	***212***	***3*.*4***	***6299***

Table 3 shows that less than half of respondents (46.2%) knew that oxytocin should be stored in the refrigerator, while more respondents incorrectly stated that oxytocin should be stored on the drug shelf (58.6%) or in a dark room (9.4%). Most doctors (71.2%) knew that oxytocin should be stored in the refrigerator, compared to nurses/midwives (46.6%) and CHWs (28.4%). A higher percentage of healthcare providers with less than 10 years of working experience (51.4%) had proper knowledge that oxytocin should be stored in the refrigerator compared to those with 10 or more years of experience (40.8%). Also, a greater proportion of respondents in private sector health facilities (52.8%) had proper knowledge that oxytocin should be stored in the refrigerator compared to those working in public health facilities (40.0%).

**Table 3:** Respondents’* characteristics by their knowledge about storage of oxytocin.

Background Characteristics		Knowledge about oxytocin storage						
		Refrigerator		On the drug shelf		In the dark		Total
		N	%	N	%	N	%	N
**Profession**	Doctor	840	71.2	415	35.2	121	10.3	1179
	Nurse/midwife	1567	46.6	1995	59.3	299	8.9	3362
	CHW	500	28.4	1281	72.9	171	9.7	1758
**Years of experience**	<10yrs	1630	51.4	1746	55.0	242	7.6	3172
	= >10yrs	1277	40.8	1945	62.2	349	11.2	3127
**Health facility type**	Public	1311	40.0	2086	63.7	297	9.1	3274
	Private	1596	52.8	1605	53.1	294	9.7	3025
**Overall**		***2907***	***46*.*2***	***3691***	***58*.*6***	***591***	***9*.*4***	***6299***

* Multiple response question.

**Fig 1:**
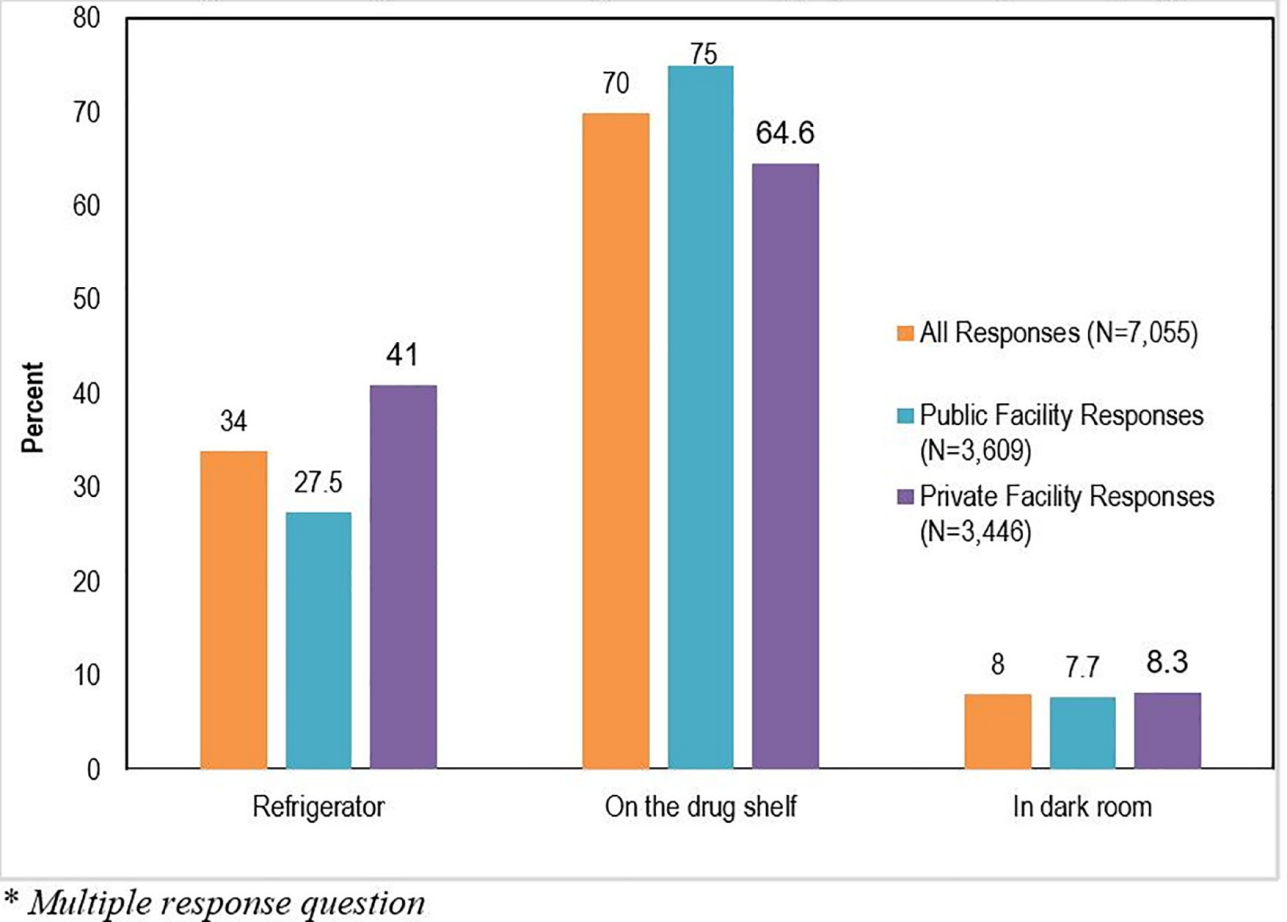
Respondents’* oxytocin storage practices by facility type.

The respondents (N = 6299) were asked about the different ways on how oxytocin is stored at their facility (*some respondents gave more than one response*). Fig 1 shows that about one-third (34.0%) of respondents indicated that oxytocin is stored in the refrigerator in their health facilities as required. Most respondents indicated that oxytocin is stored on the drug shelf in health facilities (70.0%) and some said in a dark room (8.0%). A higher proportion of responses (41.0%) from private health facilities reported that oxytocin is stored in the refrigerator (proper storage) in their facility, compared to only 27.5 percent of respondents from public health facilities.

### Administration of oxytocin

**Fig 2:**
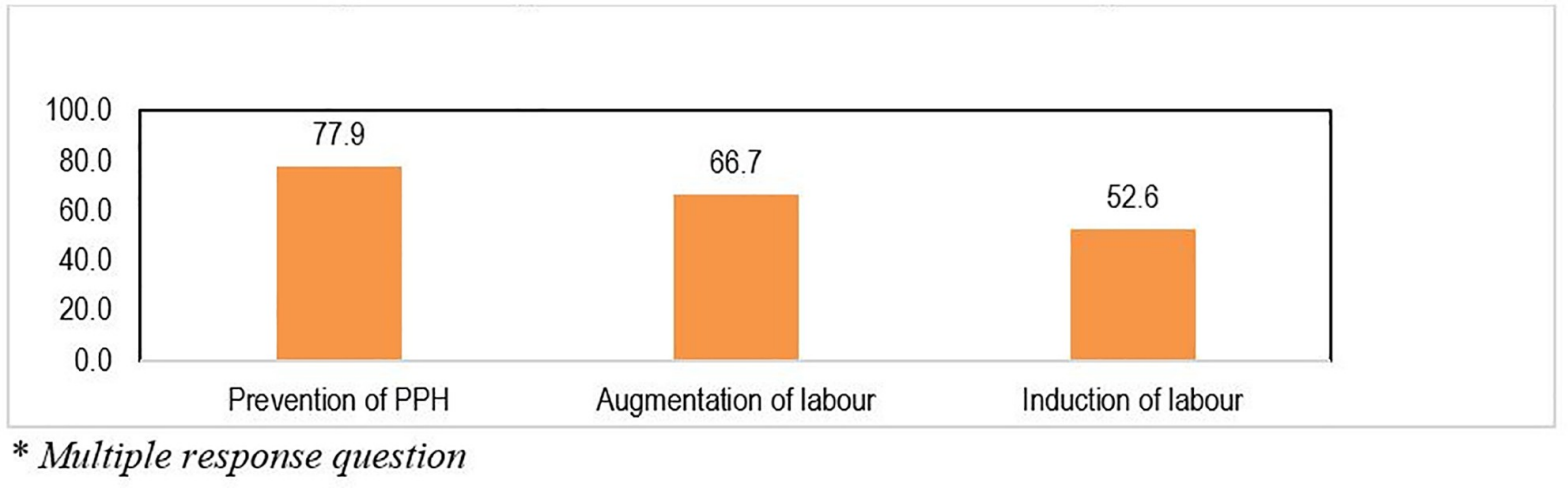
Respondents’* indication for use of oxytocin.

Fig 2 presents the administrations of oxytocin for different indications. The results show that the most common indication for the use of oxytocin by healthcare providers is for prevention of post-partum hemorrhage (77.9%) followed by augmentation of labor (66.7%). Approximately 53% of respondents said that oxytocin was used for induction of labor.

**Fig 3:**
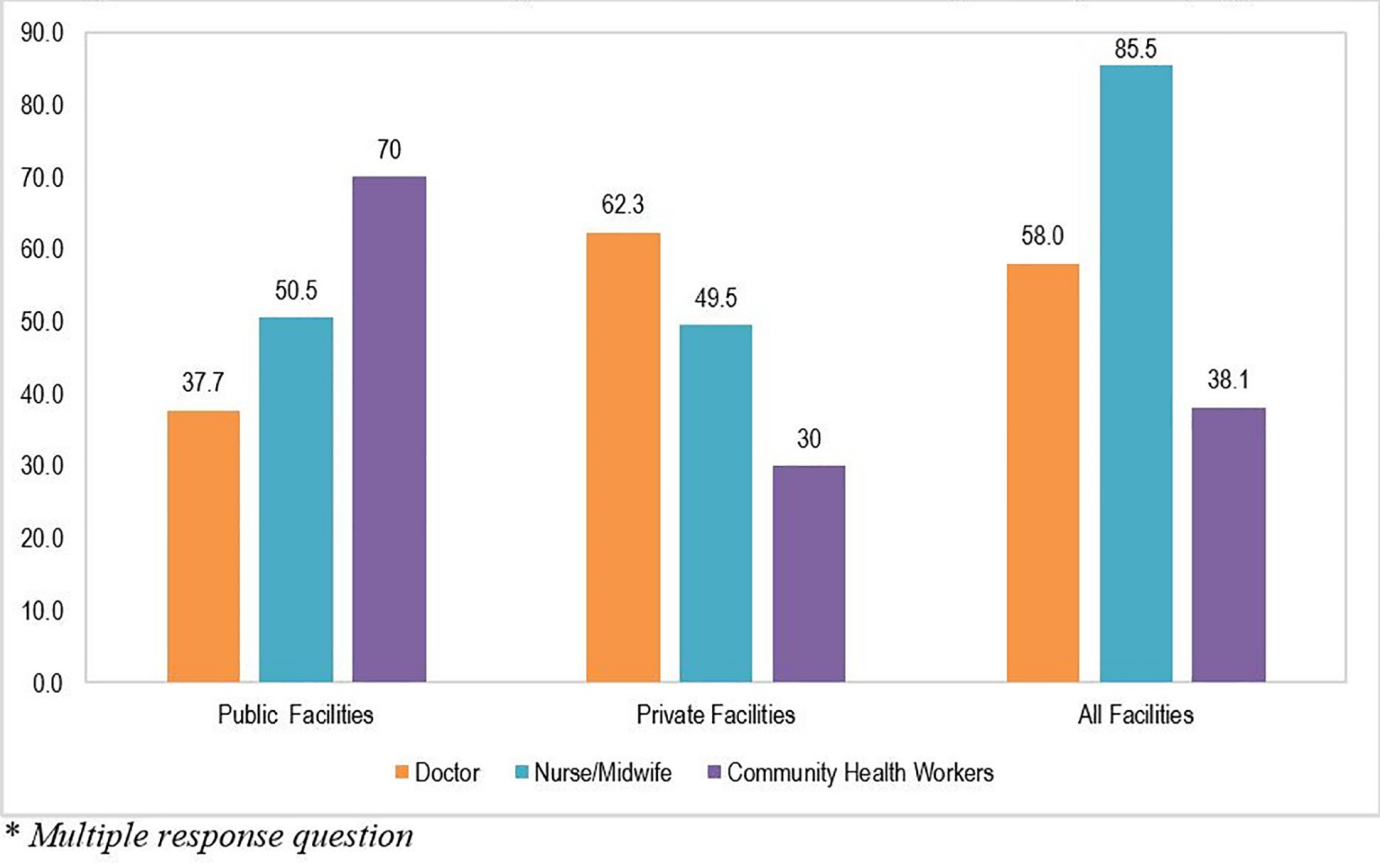
Carde of healthcare providers who administer oxytocin by facility type.

Respondents were asked as to which cadres administers oxytocin in their facility to patients. Fig 3 shows that across all facilities, oxytocin is mostly administered by nurses/midwives (85.5%), followed by doctors (58.0%) and CHWs (38.1%). Further analysis showed that a high percentage of oxytocin administrations are done by CHWs in public facilities (70%) compared to private facilities (30%) while a high percentage of oxytocin administrations in private healthcare facilities (62.3%) are conducted by doctors compared to public health facilities (37.7%).

Among respondents that reported using oxytocin for the prevention of PPH (N = 4,908), a majority of them said that they administer oxytocin to clients through intravenous infusion (76.7%) and 48.2 percent of them said they use the intramuscular route to administer oxytocin to clients. (Table 4)

**Table 4:** Respondents’* practice around route of administration of oxytocin for prevention of PPH.

Administration Route	Prevention of PPH (N = 4908)	
	*N*	*%*
**Oral**	49	1.0
**Intramuscular**	2365	48.2
**Intravenous push**	1037	21.1
**Intravenous infusion**	3765	76.7

* Multiple response question.

Table 5 shows that, of the respondents who use oxytocin for the prevention of PPH, only 42.8 percent of them use the WHO-recommended oxytocin dose of 10IU for all women. Surprisingly, a higher percentage of respondents also said that they used above WHO-recommended oxytocin dose of 10IU including 4.9 percent that used 15IU and 41.1 percent said they used 20IU or higher doses in women for prevention of PPH.

**Table 5:** Respondents’ dosage use of oxytocin for prevention of PPH in women.

Dosage of oxytocin	Prevention of PPH in Women (N = 4908)	
	*N*	*%*
**5IU**	477	9.7
**10IU**	2102	42.8
**15U**	241	4.9
**20IU or more**	2018	41.1
**Not sure**	70	1.4

Those that had used oxytocin for PPH prevention were asked whether they have ever experienced problems related to suspected or known poor-quality oxytocin used in women. Table 6 shows proportion of respondents who have experienced problems related to suspected poor quality of oxytocin used for the prevention of PPH in all women and the doses used. Slightly more than half of all respondents (53.3%) reported using 5IU or 10IU doses as recommended in Nigeria. Further analysis revealed that just over half (52.8%%) of the respondents who reported experiencing problems related to suspected poor-quality oxytocin used 15IU or higher dose compared to only (44.4%) that had not experienced oxytocin failure.

**Table 6:** Comparison of respondents’ experience of oxytocin failure by oxytocin dosage used for prevention of PPH in women.

Oxytocin dosage	Ever experienced problems related to suspected or known poor-quality oxytocin in women							
	Yes		No		Not Sure		Total	
	*N*	*% *	*N*	*%*	*N*	*%*	*N*	*%*
**5IU or 10IU**	522	47.2	1846	55.6	211	51.3	2736	53.3
**15IU/20IU/others**	585	52.8	1474	44.4	200	48.7	2113	46.7
**Total**	1109	--	3324	--	416	--	4849	--


Table 7 shows that, among those respondents who reported having experienced problems with oxytocin (N = 1339), a high percentage of them reported that their patients experienced poor uterine contraction or atony (54.8%). Hemorrhagic events in patients were reported by 35.4 percent of respondents, while maternal deaths were reported by 2.2 percent of respondents.

**Table 7:** Respondents’* who experienced failed oxytocin by type of consequences.

Type of Consequence	Respondents who experienced failed oxytocin (N = 1339)	
	*N*	*%*
**Poor uterine contractions or atony**	734	54.8
**Hemorrhage**	474	35.4
**Failed induction or augmentation**	158	11.8
**Prolonged labor**	181	13.5
**Fetal distress or IUFD**	37	2.8
**Maternal deaths**	30	2.2

* Multiple responses question.

All respondents were also asked about their knowledge on the appropriate action to take when oxytocin fails to prevent PPH in their practice (N = 6299). Table 8 shows that slightly more than half (54.3%) of respondents said that they will change to another uterotonic, while 37.1 percent said they would double the dose of oxytocin when the first administered dose of oxytocin fails to prevent PPH.

**Table 8:** Respondents’ action following oxytocin failure to prevent PPH.

Action to take	Respondents on oxytocin failure to prevent PPH (N = 6,299)	
	*N*	*%*
**Change to another uterotonic**	3421	54.3
**Double the dose**	2335	37.1
**Caesarean section**	223	3.5
**Refer**	187	3.0
**Others**	133	2.1

Table 9 shows that majority (61.6%) of the respondents told their supervisor/administrator when they perceived ineffectiveness and experienced oxytocin failure in their practice while up to 34.5 percent of respondents documented ineffectiveness of oxytocin in writing.

**Table 9:** Respondents’* action following suspected poor quality or perceived ineffectiveness of oxytocin when encountered.

Action taken	*Frequency*	*%*
Tell supervisor/administrator	4896	61.6
Document it in writing	2737	34.5
Do nothing	310	3.9
**Total**	**7943**	

* Multiple response question.

Respondents who reported ‘documenting the experience of perceived oxytocin failure or ineffectiveness’(N = 2737), were asked the channel for documentation used. Table 10 shows that more than half of the respondents documented this experience in the hospital case note while only 4.9 percent documented it in pharmacovigilance/ ADR form.

**Table 10:** Respondents’* channel for documentation of perceived ineffective oxytocin reported.

Channel	*Frequency*	*%*
Case note	1531	52.9
Hospital/Labor ward reporting system	1019	35.2
Patient chart	206	7.12
Pharmacovigilance/ ADR form	134	4.6
**Total**	**2890**	

* Multiple response question.

## Discussion

Similar to the Lagos pilot study, this study revealed poor knowledge of proper oxytocin storage despite majority of the respondents reporting that they have been previously trained on the use of oxytocin. The knowledge regarding the proper storage of oxytocin varied by cadre, years of experience, and type of facility. Most of the doctors were found to have proper knowledge compared to nurses/midwives and CHWs, which aligns with the Lagos pilot study [25]. Similarly, more respondents with less than 10 years of working experience had proper knowledge that oxytocin should be stored in the refrigerator compared to those with 10 or more years of experience. This again is similar to the findings of the Lagos study [25]. It was interesting to see that healthcare providers in private healthcare facilities have proper knowledge about correct storage of oxytocin compared to those working in public healthcare facilities. This finding was in contrast to the Lagos study, where more healthcare providers in public healthcare facilities had proper knowledge about the correct storage condition for oxytocin [27]. This difference may be due to the inclusion of CHWs in this study most of who were from public healthcare facilities without a doctor or nurse/midwives. The CHWs were not included in the Lagos study. Nevertheless, previous studies in Ghana and Nepal reported similar findings that fewer healthcare providers from public facilities have proper knowledge on the storage of oxytocin in the refrigerator [28, 32]. Other qualitative studies that assessed healthcare providers’ knowledge on oxytocin storage in other settings found that many had incorrect knowledge that oxytocin should be stored in room temperature or in the freezer [26, 32]. These findings have major programming implications in terms of raising awareness among healthcare providers that deliver babies on the importance of correct storage of oxytocin through the supply chain to maintain its effectiveness. These findings should be used to develop and revise training materials and put in place a standard protocol to guide training of healthcare providers on the use and proper storage of this life-saving commodity through intensified and continuous pre-service and in-service training.

The study revealed that in practice, oxytocin is stored on the drug shelves in the facilities as reported by most respondents. More respondents in the private facilities reported that oxytocin was stored in the refrigerator than those in the public facilities. This is not surprising given that more healthcare providers in the private facilities knew the correct storage of oxytocin than those in the public facilities. This finding suggests that healthcare providers’ knowledge regarding proper storage of oxytocin may have influenced their actual practice. It may also reflect the availability and functional refrigerators in private facilities than in public facilities. Similarly, several previous studies have reported that the common practice of oxytocin storage in facilities is at room temperature or on the drug shelf and a rare practice of oxytocin storage in the refrigerator [28, 33]. Reasons for this apparent poor practice have been reported as lack of knowledge among healthcare providers and medical storekeepers about the recommended storage condition and lack of mention or explanation of the recommended oxytocin storage conditions in their trainings or protocols in the clinics [28]. Even when healthcare providers are aware of the proper storage condition as also seen in the current study, factors beyond their control (e.g., lack of refrigerators and cold chain constraints from unreliable electricity) may affect their practices [26, 32]. Prior studies have also shown that improper storage conditions of oxytocin in the supply chain is a major contributor to the poor-quality of oxytocin used in Nigeria and other LMICs [17, 34]. In response, in December 2018 WHO issued new recommendations on other uterotonics for the prevention of PPH in tropical climates where refrigeration is limited. The recommendations included using Carbetocin (heat-stable formulation), based on evidence that it is at least as “equally effective and safe as oxytocin, and better suited for tropical climates. These efforts should be accelerated to qualify alternative medicines such as Carbetocin for the prevention of PPH in LMICs, where refrigeration is a problem”. In addition, advocacy for the adoption of WHO, UNICEF, and UNFPA joint statement made in 2019 urging stakeholders to ensure that oxytocin is managed in cold chain of 2°C–8°C for distribution and storage, and label oxytocin packages to clearly indicate storage and transport requirements [26, 32].

Oxytocin is most often administered by nurses/midwives that provide obstetrics and gynecological services in the health facilities as seen in the current study and in other previous studies [33, 35]. Yet, this study showed that many of the nurses/midwives lack proper knowledge on correct oxytocin storage. In addition, a large percentage of CHWs in public healthcare facilities administer oxytocin than in the private sector in Nigeria. These results are perhaps a reflection of the Task shifting and Task sharing policy of 2014 in Nigeria, which authorized trained CHWs to administer oxytocin, but only for the prevention of PPH and only in facilities without a skilled healthcare provider. The policy is about making more efficient use of available healthcare providers through rational redistribution of tasks among existing health workforce cadres, by moving specific tasks (where appropriate) from highly qualified providers to those with less training and fewer qualifications to improve access to health services for all Nigerian people [36]. These findings have implications for targeting training to all nurses/midwives and CHWs that offer obstetrics and gynecological services on the appropriate use of oxytocin for PPH prevention and its storage requirements in the supply chain to ensure its effectiveness.

The most common route of oxytocin administration for prevention of PPH among respondents was intravenous infusion. Oxytocin for the prevention of PPH should be administered either intravenously slow bolus or intramuscularly [10]. The intravenous route is said to have an instant effect, while the intramuscular route provides a slightly longer sustained effect [37]. Similar to a previous study, our study found that majority of our respondents used intravenous infusion as a route of administration of oxytocin followed by intramuscular route [27].

The dosing pattern of oxytocin for PPH in the current study was similar in the Lagos pilot study. Findings showed that less than half of the respondents used the WHO recommended dose (10IU) in both primiparous and multiparous women, while another half of the respondents used above the WHO-recommended dose (more than 10IU) for prevention of PPH. Specifically over a third of the respondents in the current study used 20IU (double the WHO recommended dose) while a significant proportion used greater than 20IU. Further analysis revealed that when oxytocin dose used does not work in the prevention of PPH, about half of respondents changed to another uterotonic and about a third doubled the dose of oxytocin for their patients. The Lagos pilot study found similar results where a third of respondents used doses greater than the recommended 10IU for prevention of PPH [27]. However, the respondents’ perceptions concerning the effectiveness of the oxytocin drug they use does not correlate with the reported experiences with failed oxytocin use. This finding reflects the limited awareness and knowledge among providers to suspect poor-quality of oxytocin medicine used in their practice. It was also difficult to assess this study’s findings against any standards in the country, since there is no widely available national guideline or protocol for clinical use of oxytocin during and after labor in Nigeria. The Lagos pilot study also highlighted lack of guidelines and has helped inform the ongoing advocacy for the relevant stakeholders to address this concern. Currently, most guidelines used in Nigeria are based on experiences and recommendations from the United States, Canada, and the United Kingdom, which have recommended a low dose of oxytocin (2.5IU up to 5IU) intrapartum, with much more caution when being used in multiparous women [38]. For the prevention of PPH, most guidelines and clinical practice recommend 10IU intramuscular or intravenous [10]. Previous evidence suggests that healthcare providers in Africa often use up to three vials to get the desired effect (contraction) of one vial. Often a vial of oxytocin comes in 5IU or 10IU [24]. A previous study in Karnataka, India, documented similar findings [39]. These findings further demonstrate the urgent need for developing evidence-based standardized guidelines for oxytocin dosage for Nigeria and other LMICs.

Respondents that had experienced oxytocin failure in preventing PPH, reported poor uterine contraction, hemorrhage, failed induction, prolonged labor, fetal distress, and sadly a couple of maternal deaths from failure of oxytocin to induce desired contraction in the patients.

Suspicion and reporting of therapeutic ineffectiveness is one of the scopes of pharmacovigilance and the recommendation is to document this in the pharmacovigilance form (individual case safety report-ICSR). Our findings showed that about a third of the respondents documented their perceived ineffectiveness of the oxytocin used. Of this, only 4.6 percent documented it in the pharmacovigilance form and this is similar to the findings of the Lagos study [27]. Reporting through the appropriate channel is important as this may have generated signal to a possible issue around quality of oxytocin used.

### Strengths and limitations

The major strength of this study is the large and representative sample of respondents from different cadres of healthcare providers (doctors, nurse/midwives, and CHWs) across the six geopolitical zones of Nigeria. These respondents were selected from public and private healthcare facilities which ensured the representativeness of the practices at all levels of the country’s healthcare system (tertiary/secondary/primary), hence, providing the much-needed data to inform programming and policy change in preventing PPH in Nigeria and other LMICs with high maternal mortality rates.

The study has two limitations. Since the responses were self-reported, this potentially introduced recall bias on past events. A qualitative aspect to complement the quantitative data was not conducted to help probe further on concerns around use of oxytocin during labor and delivery. The sample targets in some of the northern states were not reached due to fewer qualifying facilities that offer obstetrical and gynecological services.

## Conclusion and recommendations

The healthcare providers who help deliver babies are in a better position to unveil quality concerns of the medicines they use given that they are at the tail end of the supply chain and logistics management of medicines. The current study provides additional evidence which was not assessed in the Lagos study that despite relatively high proportion of respondents receiving training in the proper storage of oxytocin across cadres, in practice a very high proportion of respondents reported continued inappropriate storage of oxytocin in their facilities (mainly on the drug shelf), which compromises the quality of oxytocin.

This evidence calls for key stakeholders to take urgent actions on the management of cold chain constraints and other steps along the supply chain in terms of ensuring quality manufacturing, organized procurement and distribution, and guidelines for oxytocin storage at the facility level. It is also recommended that beyond development of clinical guidelines and regulation on the use of oxytocin, there is need for the dissemination and the use to continuously train healthcare providers especially nurses/midwives. This is the critical first step needed to create awareness and ensure adherence to the recommended standards in terms of oxytocin dosage, storage along the supply chain, quality concerns, and the appropriate actions when oxytocin fails to prevent PPH.

The training should ensure that all healthcare provider cadres have a clear understanding of their scope on the use of oxytocin for different indications including limitations for using oxytocin in intrapartum. In addition, regular post-marketing surveillance of quality of medical products including oxytocin should be conducted in Nigeria and other LMICs to save lives. Finally, necessary conditions (e.g., functional refrigerators, access to reliable electricity, knowledgeable supply-chain partners) to appropriately store oxytocin must be put in place. Hence, the Nigerian Government or other LMICS should provide alternative sources of energy to ensure availability of electricity especially in the remote public health facilities. These factors are completely outside the control of the healthcare provider, yet they are key in improving storage and maintaining the quality of oxytocin.

Since the 2018 WHO has new recommendation for use of Carbetocin as an alternative uterotonics for the prevention of PPH in tropical climates where refrigeration is limited. It is recommended that the Nigeria government should encourage the availability of this alternative for the prevention of PPH in Nigeria women.

Finally, there is also need for continuous training around pharmacovigilance among health workers. This is to increase their awareness and practice around the suspicion and reporting of therapeutic ineffectiveness which is also within the scope of pharmacovigilance.

## Supporting information

S1 Fig Respondents’* oxytocin storage practices by facility type. (TIFF)

S2 Fig Respondents’* indication for use of oxytocin. (TIFF)

S3 Fig Cadre of healthcare providers who administer oxytocin by facility type. (TIFF)

S1 File Data set. (XLSX)

S2 File Questionnaire for oxytocin survey. (DOCX)

S3 File Respondents’ informed consent form. (DOCX)
