# Supplementary figures and images for: A descriptive study of healthcare-providers’ experiences with the use and quality of oxytocin for the prevention of post-partum hemorrhage in Nigeria: A nation-wide survey
Source: PLoS One. 2021 Oct 6;16(10):e0258096. doi: 10.1371/journal.pone.0258096 (PMC8494301; doi:10.1371/journal.pone.0258096)

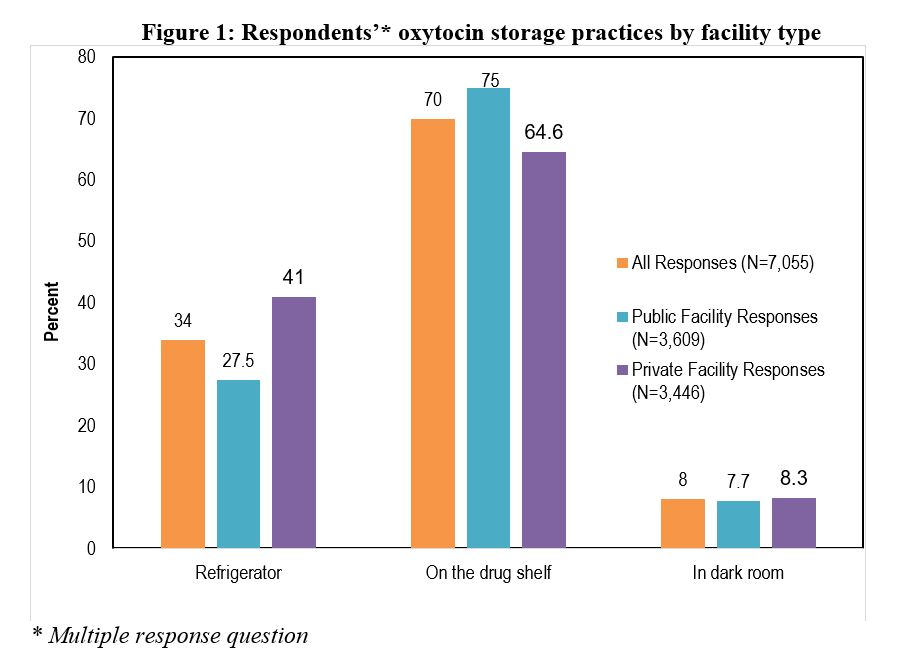

Supplement: S1 Fig — (TIFF) [file pone.0258096.s001.tiff]

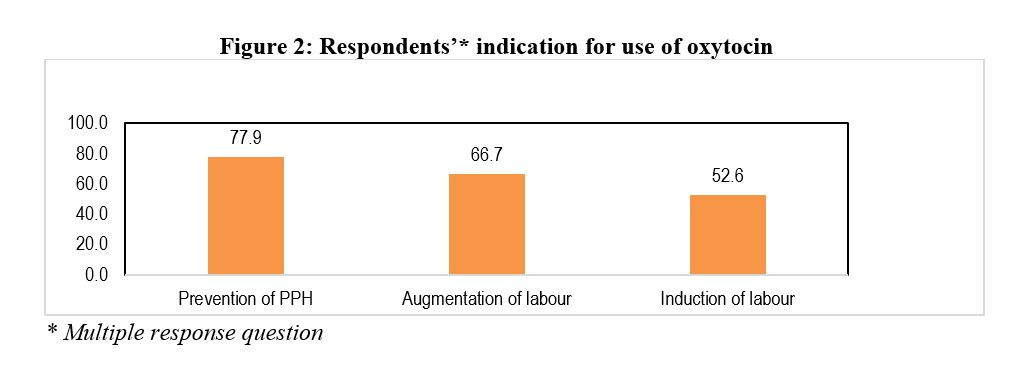

Supplement: S2 Fig — (TIFF) [file pone.0258096.s002.tiff]

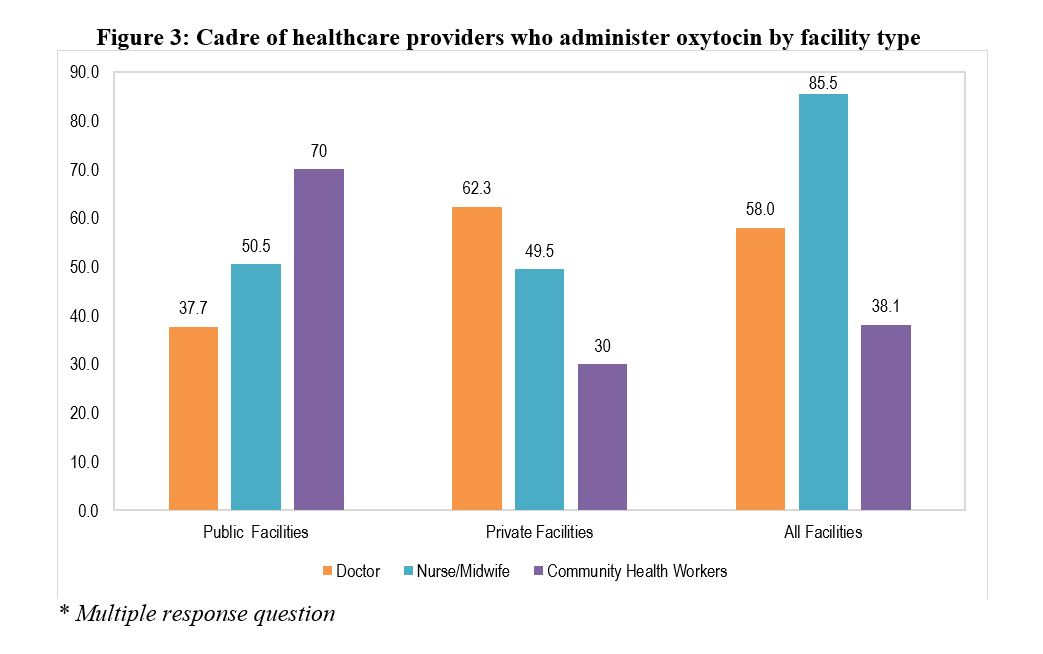

Supplement: S3 Fig — (TIFF) [file pone.0258096.s003.tiff]
