# Supplementary material for: A descriptive study of healthcare-providers’ experiences with the use and quality of oxytocin for the prevention of post-partum hemorrhage in Nigeria: A nation-wide survey
Source: PLoS One. 2021 Oct 6;16(10):e0258096. doi: 10.1371/journal.pone.0258096 (PMC8494301; doi:10.1371/journal.pone.0258096)
Supplement: S2 File — (DOCX) [file pone.0258096.s005.docx]

**CLINICAL EXPERIENCES OF OXYTOCIN QUALITY USED BY HEALTH CARE PROVIDERS (WHO TAKE DELIVERIES)**

| **Research Assistant Name:** | **Respondent’s Initials:** |
| --- | --- |
| **Date of Interview:** | **LGA:** |
| **Interview #:** |  |
| The goal of the study is to understand the pattern of care provided to pregnant women during childbirth and immediately postpartum with the use of oxytocin. We hope to develop recommendations for the Ministry of Health, doctors, nurses and other healthcare providers to improve knowledge and practices in the use of oxytocin in public or private facilities that offer obstetrics and gynecological services. There is no right or wrong answer. We just want to know what your practices and experiences are. All information collected in this study will be coded and no names will be recorded. The information cannot be linked to you in anyway and your name, facility name or any other identifier will not be used in any publication or reports from this study. | |

| **No.** | **Question** | **Coding Category** | |
| --- | --- | --- | --- |
| **Instructions: Please read the questions carefully and provide your answer as honestly as possible that most describes your experience or state and use the blank space to write in answers for the “Other” category. Circle the most appropriate answer.** | | | |
| **SECTION A: SOCIO-DEMOGRAPHICS OF RESPONDENTS** | | | |
| 1 | Age at last birthday: | _____________________ |  |
| 2 | Gender: | Male  Female | 1  2 |
|  |  |  |  |
| **SECTION B: OCCUPATIONAL HISTORY OF RESPONDENTS** | | | |
| 3 | Occupation: | Doctor  Nurse/midwife  Community health workers  Other: ___________________ | 1  2  3  66 |
| 4 | Years of working experience : | _________________________ |  |
| 5 | Type of health facility: | Government  Private  Non-profit | 1  2  3 |
|  |  |  |  |
| **SECTION C: CLINICAL EXPERIENCE WITH OXYTOCIN** | | |  |
| 6 (a) | Have you ever used oxytocin in your career?  **If no, go to end of survey script. If yes, continue to question 6 (b).** | Yes  No  Not Sure | 1  2  3 |
| 6 (b) | How often do you use oxytocin? | Daily  Weekly  Monthly  Others (specify): ___________ | 1  2  3  66 |
| 7 | If yes, for what purpose(s) did you use oxytocin?  **May select more than one option** | Stimulation of labour  Augmentation of labour  Induction of labour  Prevention of post-partum Hemorrhage | 1  2  3  4 |

| 8 | Have you had any training on the use of oxytocin in labour or storage of oxytocin? | Yes  No  Not sure | 1  2  3 |
| --- | --- | --- | --- |
| 9 | What dose of oxytocin do you usually use for stimulation of labour? | 2.5IU  5IU  10IU  15IU  20IU  Not sure  Other:_________________ | 1  2  3  4  5  6  7 |
| 10 | What dose of oxytocin do you usually use for augmentation of labour in primiparous women (1^st^ time delivery)? | 5IU  10IU  15IU  20IU  Not sure  Other:_________________ | 1  2  3  4  5  66 |
| 11 | What dose of oxytocin do you usually use for augmentation of labour in multiparous women (more than one delivery)? | 5IU  10IU  15IU  20IU  Not sure  Other:_________________ | 1  2  3  4  5  66 |
| 12 | What dose of oxytocin do you usually use for induction of labour in primiparous women (1^st^ time delivery)? | 5IU  10IU  15IU  20IU  Not sure  Other:_________________ | 1  2  3  4  5  66 |
| 13 | What dose of oxytocin do you usually use for induction of labour in multiparous women (more than one delivery)? | 5IU  10IU  15IU  20IU  Not sure  Other:_________________ | 1  2  3  4  5  66 |
| 14 | What dose of oxytocin do you usually use for prevention of postpartum hemorrhage of labour in primiparous women (1^st^ time delivery)? | 5IU  10IU  15IU  20IU  Not sure  Other:_________________ | 1  2  3  4  5  66 |
| 15 | What dose of oxytocin do you usually use for prevention of postpartum hemorrhage of labour in multiparous women (more than one delivery)? | 5IU  10IU  15IU  20IU  Not sure  Other:_________________ | 1  2  3  4  5  66 |

| 16 | Which route of oxytocin administration do you use for the stimulation of labour?  **May select more than one option** | Oral  Intramuscular  Intravenous push  Intravenous infusion  Posterior  Not sure  Other:_________________ | 1  2  3  4  5  6  66 |
| --- | --- | --- | --- |
| 17 | Which route of oxytocin administration do you use in the induction of labour?  **May select more than one option** | Oral  Intramuscular  Intravenous push  Intravenous infusion  Posterior  Not sure  Other:_________________ | 1  2  3  4  5  6  66 |
| 18 | Which route of oxytocin administration do you use in augmentation of labour?  **May select more than one option** | Oral  Intramuscular  Intravenous push  Intravenous infusion  Posterior  Not sure  Other:_________________ | 1  2  3  4  5  6  66 |
| 19 | Which route of administration of oxytocin do you use in the prevention of postpartum hemorrhage?  **May select more than one option** | Oral  Intramuscular  Intravenous push  Intravenous infusion  Posterior  Not sure  Other:_________________ | 1  2  3  4  5  6  66 |
| 20 | Which cadre of health care provider administers oxytocin in your facility?  **May select more than one option** | Doctor  Nurse/Midwife  Community health workers  Other | 1  2  3  66 |
| 21 | When you administer oxytocin to a patient during labour, what do you monitor to see if the drug is working?  **May select more than one option** | 3 adequate uterine contractions of 40 to 60 secs in 10 minutes  Cervical dilatation  Not sure  Other:____________________________________________ | 1  2  3  66 |
| 22 | Which brand(s) of oxytocin do you use often in the last one year?  **May select more than one option** | Pitocin  Syntocinon  Labtocin  Oxitocin  Other: ___________________  Other: ___________________  Other: ___________________  Other: | 1  2  3  4  66  67  68  69 |

| 23 | Of the following drugs, do you think these drugs are effective in helping your patients? (Effectiveness of oxytocin in this study is the ability of the oxytocin injection used, to achieve the desired contraction within the recommended dose for a specific indication.)  (NB. The real oxytocin brands were de-identified for the purpose of sharing this tool) | A  B  C  D  Other:-------  Other:-------  Other:-------  Other:------- | \| **Effective**  1  1  1  1  1  1  1  1 \| **Ineffective**  2  2  2  2  2  2  2  2 \| **Unsure**  3  3  3  3  3  3  3  3 \| \| --- \| --- \| --- \| | |
| --- | --- | --- | --- | --- | --- | --- | --- |
| 24 | In most cases, does the clinic/hospital facility or the patient purchase (or acquire) the oxytocin? | Patient  Hospital/Clinic Facilities  Other:_________________ | | 1  2  66 |
| 25 | What is your understanding of how oxytocin is supposed to be stored?  **May select more than one response.** | In the refrigerator  On the drug shelf  In the dark  Other:____________________ | | 1  2  3  66 |
| 26 | How is oxytocin stored at this facility?  (your response may be due to circumstance beyond your control)  **May select more than one response.** | In the refrigerator  On the drug shelf  In the dark  Other:____________________ | | 1  2  3  66 |
| 27 | If the oxytocin is not working in your patient for prevention of post-partum hemorrhage, what do you do?  **If the response is “change the drug” go to question 28, if not, go to question 29.** | Double the dose  Change the drug  Caesarean section  Other:__________________ | | 1  2  3  66 |
| 28 | If you change drugs, which drug did you change to? | Ergometrine  Misoprostol  Dinoprostone  Carbetocin  Carboprost  Traxenamic acid  Other:___________________ | | 1  2  3  4  5  66  67 |
| 29 | Have you ever experienced problems related to suspected or known poor quality oxytocin?  **If Yes, go to question 30. If No, go to question 31.** | Yes  No  Not Sure | | 1  2  3 |
| 30 | What consequences or implications of poor oxytocin quality you have seen or experienced in your clinical practice? | _______________________________________ | | |

| 31 | What would you do if you suspect oxytocin used was ineffective or a drug was of poor quality (i.e., fake, substandard, expired, etc.)?  **May select more than one response.** | Tell supervisor/administrator  Document it in writing  Do nothing  Other:___________________ | 1  2  3  66 |
| --- | --- | --- | --- |
| 32 | If you document it in writing, please indicate where you write it down/document it (as per Q31) | ______________________________________ | |
| 33 | Do you have any recommendations for how to improve health care providers’ knowledge, use or storage of oxytocin in health facilities? |  | |
|  | **End of Survey Script:** Thank you for participating in our survey. We hope to use this information to advocate for better availability and use of quality-assured medicines to prevent maternal mortality. We appreciate your time. | | |
|  |  |  |  |
|  |  |  |  |
