# Supplementary material for: A descriptive study of healthcare-providers’ experiences with the use and quality of oxytocin for the prevention of post-partum hemorrhage in Nigeria: A nation-wide survey
Source: PLoS One. 2021 Oct 6;16(10):e0258096. doi: 10.1371/journal.pone.0258096 (PMC8494301; doi:10.1371/journal.pone.0258096)
Supplement: S3 File — (DOCX) [file pone.0258096.s006.docx]

**RESPONDENTS INFORMED CONSENT FORM**

**NHREC ASSIGNED NO: 01/01/2007**

**Title of Research:** Clinical experiences with oxytocin quality used by healthcare providers (who take delivery)

**Name & Affiliation of Researcher:** The principal investigator is Dr. Chioma Ejekam of the Department of Community Health, Lagos University Teaching Hospital, supported by other team of researchers.

**Funding:** Project is funded by Merck Sharp & Dohme (MSD) through Merck for Mothers initiative and implemented by United States Pharmacopeia (USP).

**Introduction:** Post-partum haemorrhage (PPH) is a leading cause of maternal mortality in Nigeria and most low-income countries and it is preventable. The World Health Organization (WHO) has recommended oxytocin an effective and safest drug of first choice in the prevention and treatment of PPH in the active management of the third stage of labour for improving the health of women during childbirth. However, there are concerns about its quality.

**Purpose of research:** This research hopes to assess the clinical experience of healthcare providers (who take deliveries) with the quality of oxytocin encountered in their practice.

**Procedure of the research:** The questionnaire is self-administered and will obtain information on your occupational history, knowledge of oxytocin and clinical experiences with the quality of oxytocin often used in your practice. It takes about 10 minutes to fill the questionnaire.

**Potential benefit(s):** The study may unveil the possible contribution of poor quality oxytocin to maternal morbidity and mortality statistics in Nigeria and ultimately improve patient’s safety. There is no monetary benefit from participation.

**Willingness to participate:** Your participation in this research is entirely voluntary. The data obtained does not constitute part of your employment records.

**Potential Risks:** The research is questionnaire-based and the respondents are not considered a vulnerable population hence no harm is anticipated.

**Confidentiality:** All information obtained in this research will be coded and no names will be recorded. The information cannot be linked to you in anyway hence cannot be used in any publication or reports from this research. The results from this research may be presented at scientific or medical meetings or published in scientific journals.

**Statement of person giving consent:**

I have read the description of the research and I understand that my participation is voluntary. I know enough about the purpose, methods, risks and benefits of the research study to judge that I want to take part in it.

SIGNATURE_________________________ DATE___________________

**For further enquiry, please contact:**

Dr. C. Ejekam

bfchioma@yahoo.com
